# A Two-Staged Surgical Approach to Postero-Lateral Knee Dislocation With Bicruciate Ligament Reconstruction: A Case Report

**DOI:** 10.7759/cureus.102053

**Published:** 2026-01-22

**Authors:** Filipe Maçães, Pedro Seabra, João Dinis, Catarina Aleixo, Ricardo Pereira

**Affiliations:** 1 Department of Orthopedics and Traumatology, Local Health Unit of Gaia and Espinho, Vila Nova de Gaia, PRT

**Keywords:** bicruciate ligament reconstruction, knee dislocation, multiligament knee injury, posterolateral knee dislocation, staged surgical reconstruction

## Abstract

This paper details a novel two-staged surgical approach to managing postero-lateral knee dislocations, a rare and challenging clinical scenario. Our case study highlights the successful application of a bicruciate reconstruction of both the anterior cruciate ligament (ACL) and posterior cruciate ligament (PCL), which has not been extensively documented in existing medical literature. The treatment protocol initiated with urgent surgical reduction followed by a delayed reconstruction, allowing for initial soft tissue stabilization. This method provided significant functional recovery, enabling the patient to resume daily activities with satisfactory joint mobility. The outcomes of this unique surgical strategy demonstrate its effectiveness and the potential for broader application, suggesting that staged bicruciate reconstruction may represent a valuable option in selected cases of complex knee dislocation. This case contributes a new perspective to the orthopedic field, advocating for further research into staged reconstruction techniques.

## Introduction

Knee dislocations represent a rare yet serious traumatic injury with significant potential for vascular and neural complications [1]. They comprise less than 0.01% of all trauma cases and have been reported to result in vascular injuries in up to 65% of cases involving high-velocity trauma [2,3].

Schenck et al. proposed a biomechanical classification based on the pattern of ligament injury, which was later refined by Wascher et al. to include associated vascular, neurological, or osseous lesions. This revised Schenck classification is now widely adopted in clinical settings [4].

Postero-lateral knee dislocations, a subtype of knee dislocations, are characterized by an irreducible knee due to the interposition of medial soft tissue within the femoro-tibial joint [5]. Such rotatory dislocations, though rare, constitute the majority of irreducible knee dislocations [6]. Urgüden et al. suggested that in postero-lateral dislocations, further displacement of the knee is prevented by the invagination of medial capsular and retinacular structures, which also reduce the risk of arterial injury [7].

Given the orthopedic emergency nature of such injuries, immediate surgical intervention for open reduction is imperative. After initial stabilization and rehabilitation, the necessity for elective reconstruction of the anterior cruciate ligament (ACL) or posterior cruciate ligament (PCL), or both, remains a subject of clinical decision-making [8].

Following the case reporting guidelines (CARE checklist), this report describes a case of irreducible postero-lateral knee dislocation managed with a staged surgical approach and bicruciate ligament reconstruction [9].

## Case presentation

**Figure 1:**
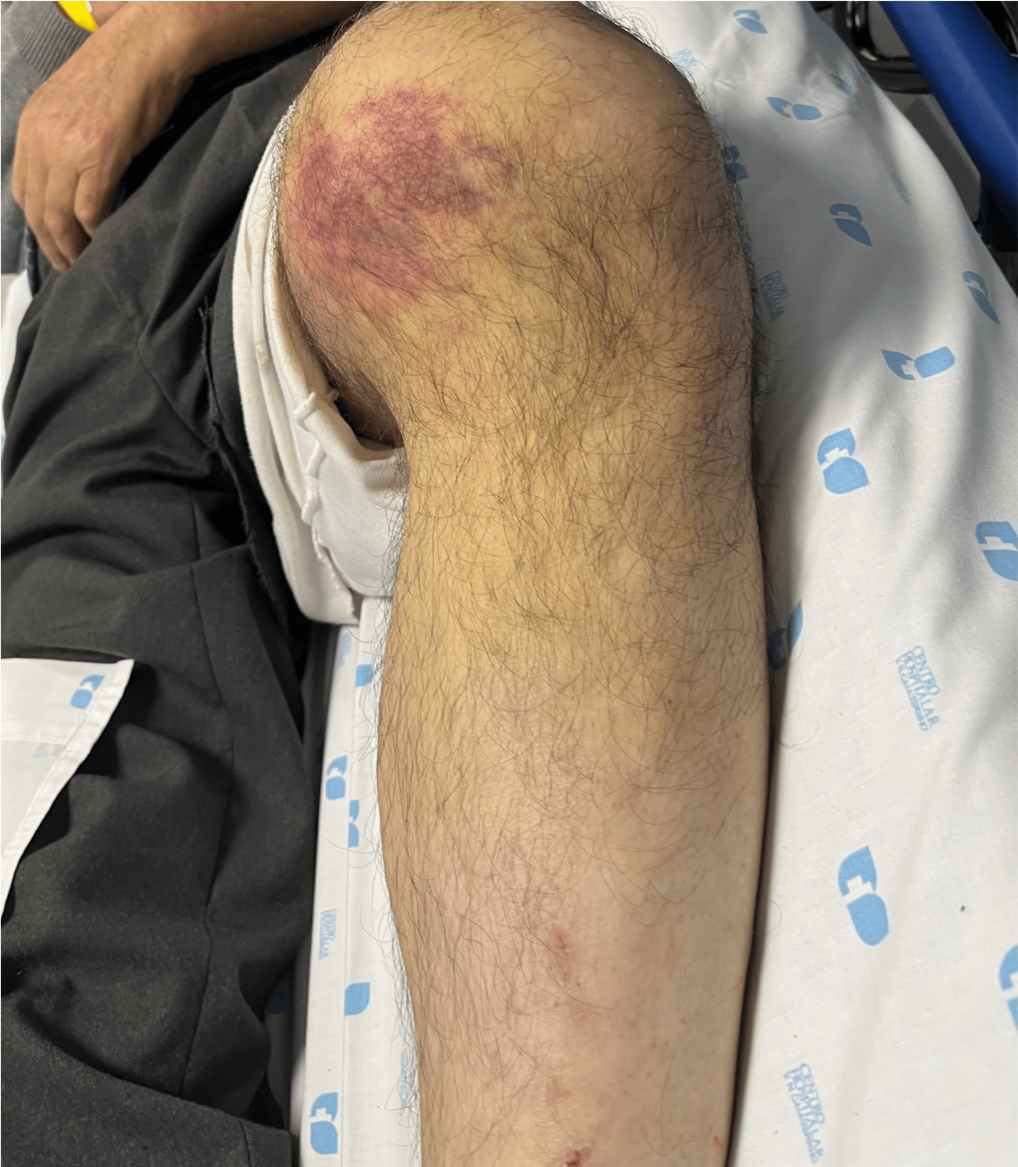
Clinical appearance of postero-lateral knee dislocation Clinical photograph of the left knee demonstrating medial ecchymosis and deformity following high-energy trauma, consistent with a postero-lateral knee dislocation.

**Figure 2:**
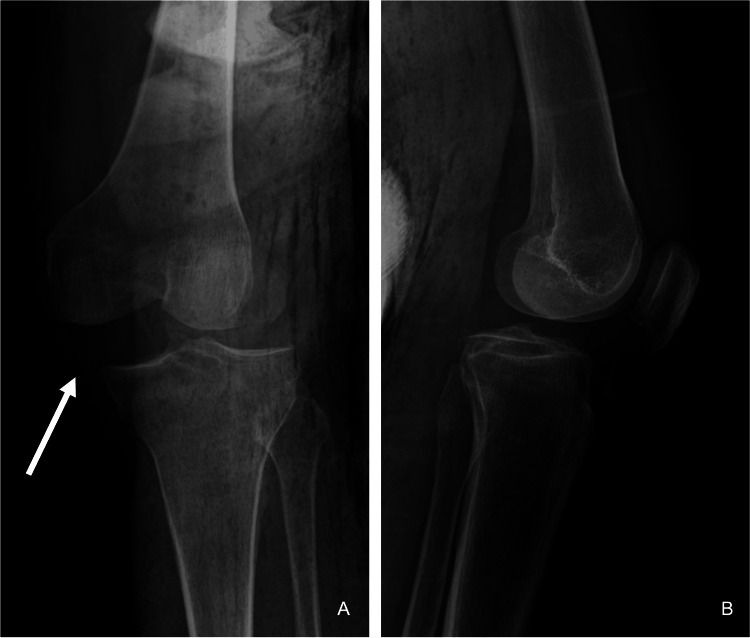
Pre-reduction radiographs of postero-lateral knee dislocation A: anteroposterior; B: lateral radiographs of the left knee showing postero-lateral dislocation with lateral displacement and rotational malalignment of the tibia relative to the femur (marked by a white arrow).

A 73-year-old male with a history of hypertension and dyslipidemia presented to our emergency department following a high-energy trauma caused by a farm animal impacting his left knee. Initial examination revealed a knee deformity and medial ecchymosis, indicative of a postero-lateral knee dislocation (Figure 1). Peripheral pulses were palpable, and the foot was warm. After an unsuccessful attempt at closed reduction, subsequent radiographic imaging confirmed a postero-lateral knee dislocation with lateral displacement and rotation of the tibia relative to the femur (Figure 2). The medial opening of the femoral-tibial joint suggests interposition of soft tissue. CT angiography excluded vascular injuries.

**Figure 3:**
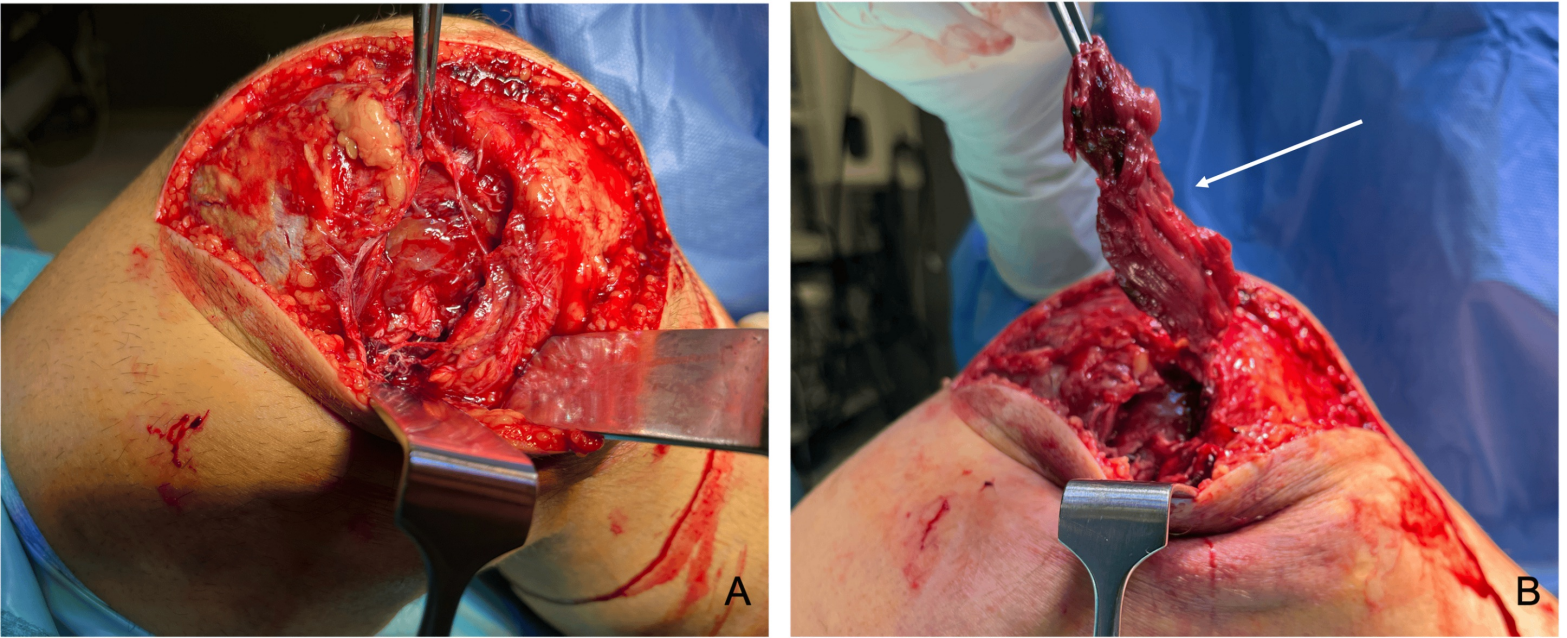
Intraoperative findings during open reduction Intraoperative photographs obtained during emergent open reduction of a postero-lateral knee dislocation. A: rupture of the medial capsular and retinacular structures, including the medial collateral ligament; B: vastus medialis muscle previously entrapped within the femorotibial joint, preventing closed reduction (marked by a white arrow).

During the urgent surgical intervention, a standard medial parapatellar arthrotomy was performed. We discovered a rupture of the medial capsule, medial retinaculum, and medial collateral ligament (MCL), with part of the vastus medialis muscle entrapped in the joint (Figure 3). We repaired the femoral insertion of the MCL with suture anchors and directly repaired the medial capsule and retinaculum. Given the initial instability and soft tissue injury, cruciate ligament reconstruction was intentionally deferred. The knee was then fixed with a knee brace, and the patient began partial weight bearing and knee mobilization 15 days postoperatively.

**Figure 4:**
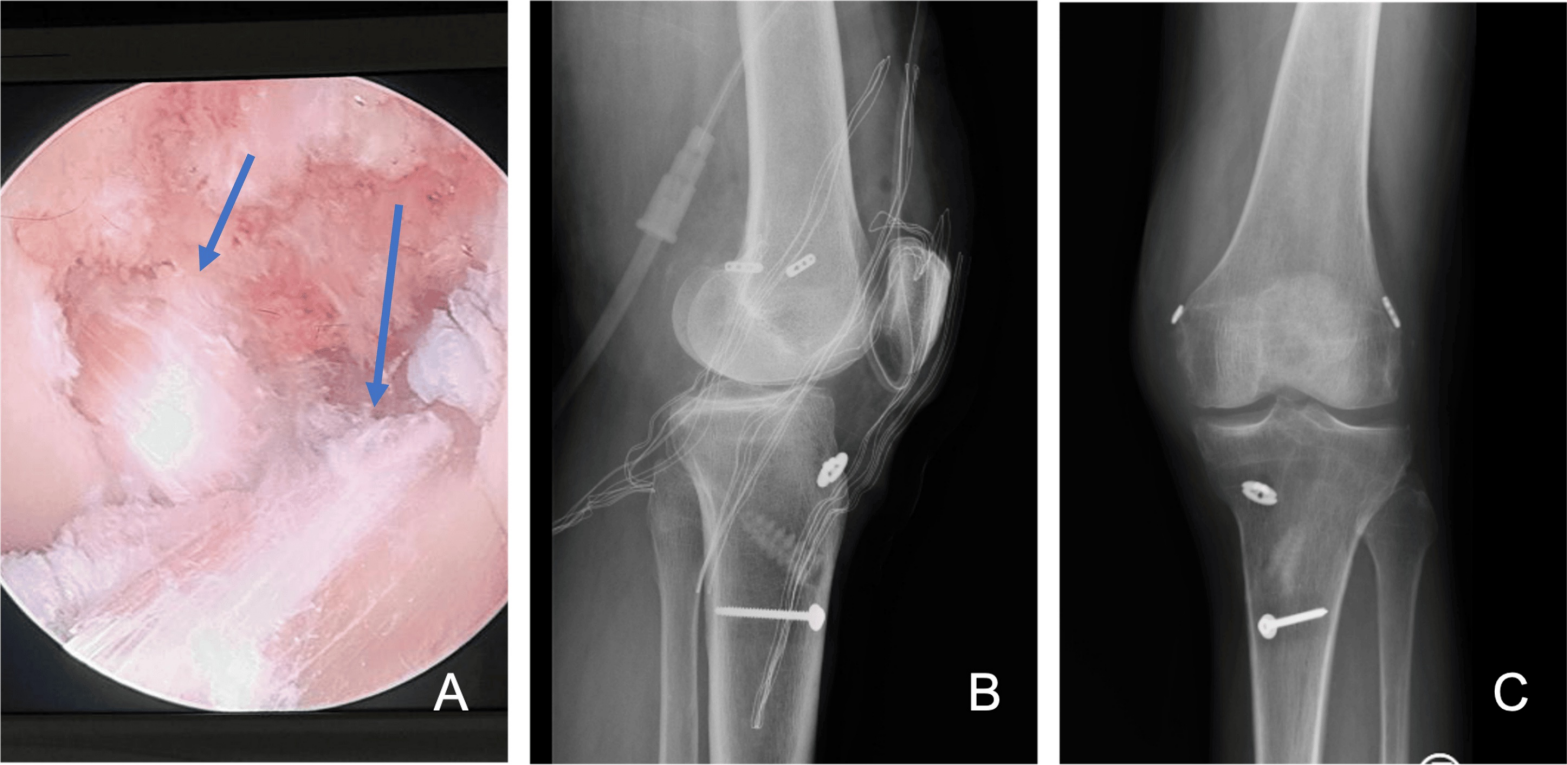
Arthroscopic and postoperative imaging following bicruciate reconstruction Intraoperative and postoperative imaging following second-stage bicruciate ligament reconstruction. A: arthroscopic view demonstrating completed anterior and posterior cruciate ligament reconstructions (marked by blue arrows); B: anteroposterior; and C: lateral radiographs of the left knee confirming appropriate tunnel positioning and graft fixation.

Post-operatively, the patient was immobilized with a knee brace, starting partial weight bearing and knee mobilization 15 days after surgery. Subsequent MRI imaging revealed complete ruptures of both the ACL and PCL, and the patient exhibited clinical signs of instability. Four months after the initial surgical reduction, and given the patient’s high functional demands and desire to maintain independent ambulation, we proceeded with bicruciate ligament reconstruction. Diagnostic arthroscopy performed at the beginning of the second-stage procedure confirmed a horizontal tear of the medial meniscus, which was repaired using an all-inside technique. An outside-in ACL reconstruction was then performed using a semitendinous autograft and a PCL inside-out reconstruction using a tibialis anterior autograft (Figure 4).

The rehabilitation protocol played a crucial role in the patient's recovery, enabling him to resume daily activities. Rehabilitation progression was individualized based on pain tolerance, knee stability, and range of motion, with gradual advancement of weight-bearing and strengthening exercises. At the six-month follow-up, the patient achieved a 0-100° range of motion, with negative anterior and posterior drawer tests, and reported satisfaction with the functional recovery of his knee.

## Discussion

A postero-lateral knee dislocation is an uncommon entity and typically results from a combination of valgus force applied to a semi-flexed knee with a rotation of the tibia relative to the femur. There remains no consensus on the precise rotational direction of the tibia associated with these injuries [8]. Typically linked with high-energy trauma such as vehicular accidents or sports injuries, these dislocations can also occur under low-energy conditions [6].

In clinical evaluations, the presence of an anteromedial distal thigh dimple sign should prompt a high index of suspicion for a postero-lateral knee dislocation, as this feature is considered pathognomonic for this type of dislocation. Closed reduction should not be attempted, as they are irreducible. Plain radiographs will be unable to reveal a true anteroposterior (AP) or lateral view of both the tibia and the femur in any single radiograph [10]. Initial evaluation should prioritize limb vascularity. While some authors perform CT angiography for every postero-lateral knee dislocation, Mills et al. demonstrated that an ankle brachial index (ABI) < 0.9 had a sensitivity, specificity, and positive predictive value of 100%, and an ABI > 0.9 had a negative predictive value of 100% for arterial injury necessitating surgical intervention [11]. Therefore, our initial intervention should focus on foot pulses and temperature and measuring ABI; CT angiography is reserved for cases with suspicious findings.

The first step of the surgical treatment is emergent open reduction due to the risk of skin necrosis at the point of medial invagination. If possible, after reduction, the medial structures should be repaired, and the knee can be fixed either with a hinged knee brace or external fixation [8]. The follow-up should include gradual mobilization of the knee and rehabilitation programs. MRI can be obtained following a period of soft tissue healing in patients with persistent symptoms. In the literature, almost all patients submitted to MRI after postero-lateral knee dislocation had bicruciate ligament injuries (ACL and PCL). Although some authors reconstructed the ACL, others didn’t reconstruct any of the ligaments, and no published reports describe staged reconstruction of both cruciate ligaments following postero-lateral knee dislocation [8]. Given the absence of a consensus regarding the timing and necessity of cruciate ligament lesion repair, the decision-making process must be individualized, taking into account factors such as the patient's age, daily activity levels, and pre-existing degenerative changes in the knee [12]. There is evidence that suggests better outcomes for surgical stabilization of ligamentous tears compared with conservative treatment after a knee dislocation [13].

When deciding to reconstruct one or both cruciate ligaments, the ongoing debate concerns whether to perform the reconstruction simultaneously with the open surgical reduction in a single-stage procedure or to opt for a delayed reconstruction approach [14]. Additional considerations influencing this decision include the availability of an experienced knee surgeon and the requisite surgical materials, given the emergent nature of the open reduction procedure. The staged approach allows recovery of medial soft tissues, reduces capsular tension, and facilitates graft handling during cruciate ligament reconstruction.

In our case, we initially opted to stabilize the knee and address the MCL, medial capsular, and retinacular ruptures. During outpatient follow-up, the patient was re-evaluated, and persistent instability was confirmed, significantly impacting activities of daily living and limiting independent ambulation. Based on clinical findings and patient-reported functional limitation, the decision was made to proceed with bicruciate ligament reconstruction in order to restore knee stability and optimize anatomic joint biomechanics.

A limitation of this case report is the absence of pre- and post-intervention validated functional outcome scores; however, functional improvement was assessed through objective clinical examination and patient-reported outcomes during follow-up.

## Conclusions

This article presents a novel two-staged surgical approach for the management of postero-lateral knee dislocations, an uncommon and complex injury that poses significant challenges in orthopedic surgery. Our case involved a bicruciate reconstruction with both ACL and PCL ligaments, a method not extensively documented in current medical literature.

The unique aspect of our approach lies in the delayed reconstruction of both cruciate ligaments. This strategy facilitated targeted treatment of the ligamentous injuries and optimized soft tissue recovery, leading to satisfactory functional outcomes, including the ability to resume daily activities and achieve a significant postoperative range of motion. Further studies are warranted to better define the role of staged bicruciate reconstruction in the management of complex knee dislocations.
